# Insights into the CRISPR/Cas system of *Gardnerella vaginalis*

**DOI:** 10.1186/1471-2180-12-301

**Published:** 2012-12-21

**Authors:** Milda Pleckaityte, Milda Zilnyte, Aurelija Zvirbliene

**Affiliations:** 1 Institute of Biotechnology, Vilnius University, Graiciuno 8, Vilnius, LT-02241, Lithuania

**Keywords:** *Gardnerella vaginalis*, Bacterial vaginosis, CRISPR/Cas, Spacer, Repeat, PAM

## Abstract

**Background:**

*Gardnerella vaginalis* is identified as the predominant colonist of the vaginal tracts of women diagnosed with bacterial vaginosis (BV). *G. vaginalis* can be isolated from healthy women, and an asymptomatic BV state is also recognised. The association of *G. vaginalis* with different clinical phenotypes could be explained by different cytotoxicity of the strains, presumably based on disparate gene content. The contribution of horizontal gene transfer to shaping the genomes of *G. vaginalis* is acknowledged. The CRISPR loci of the recently discovered CRISPR/Cas microbial defence system provide a historical view of the exposure of prokaryotes to a variety of foreign genetic elements.

**Results:**

The CRISPR/Cas loci were analysed using available sequence data from three *G. vaginalis* complete genomes and 18 *G. vaginalis* draft genomes in the NCBI database, as well as PCR amplicons of the genomic DNA of 17 clinical isolates. The *cas* genes in the CRISPR/Cas loci of *G. vaginalis* belong to the E. coli subtype. Approximately 20% of the spacers had matches in the GenBank database. Sequence analysis of the CRISPR arrays revealed that nearly half of the spacers matched *G. vaginalis* chromosomal sequences. The spacers that matched *G. vaginalis* chromosomal sequences were determined to not be self-targeting and were presumably neither constituents of mobile-element-associated genes nor derived from plasmids/viruses. The protospacers targeted by these spacers displayed conserved protospacer-adjacent motifs.

**Conclusions:**

The CRISPR/Cas system has been identified in about one half of the analysed *G. vaginalis* strains. Our analysis of CRISPR sequences did not reveal a potential link between their presence and the virulence of the *G. vaginalis* strains. Based on the origins of the spacers found in the *G. vaginalis* CRISPR arrays, we hypothesise that the transfer of genetic material among *G. vaginalis* strains could be regulated by the CRISPR/Cas mechanism. The present study is the first attempt to determine and analyse the CRISPR loci of bacteria isolated from the human vaginal tract.

## Background

*Gardnerella vaginalis*, a facultatively anaerobic bacterium of the *Bifidobacteriaceae* family, is strongly associated with bacterial vaginosis (BV): a disease characterised by malodorous vaginal discharge [1-3]. Women with BV are at risk of poor reproductive health outcomes and the acquisition of some sexually transmitted diseases [2,4]. BV is defined as a shift in microbial species from hydrogen peroxide producing *Lactobacillus* to anaerobic bacteria including *G. vaginalis*, *Atopobium vaginae*, *Prevotella*, *Peptostreptococcus*, and *Bacteroides* spp. [5,6]. The gold standard for laboratory diagnosis of BV is the Gram stain, which is used to determine the relative concentrations of lactobacilli and the bacteria characteristic of BV [7]. The state of asymptomatic BV has also been recognised, although Gram stains revealed a decrease in lactobacilli and an increase in the abundance of anaerobes specific to BV [8]. The same *G. vaginalis* that is recovered as the prevailing inhabitant of the vaginal tracts of women diagnosed with BV is also found in BV-negative women, though at much lower numbers [5,9,10]. The issue of *G. vaginalis* commensalism is still unclear, as the vaginal bacterial community is dynamic and tends to change during the menstrual cycle to produce transient dominance of *G. vaginalis* in healthy women [11,12]. Using culture-independent techniques, it was demonstrated that the vaginal microbiota may differ among human populations: Hispanic and non-Hispanic black women have significantly more anaerobes and fewer lactobacilli than Asian and Caucasian women [12]. Thus, low counts of *Lactobacillus* do not necessarily indicate the BV state [6,13].

The association of *G. vaginalis* with different clinical phenotypes could be explained by different cytotoxicity of the strains, presumably based on disparities in their gene content. Until recently, surprisingly little has been known about the genetics of *G. vaginalis*. In 2010, the genomes of several *G. vaginalis* strains from the vaginas of BV and non-BV patients were sequenced, providing information about the bacterium and enabling comparative genomic analyses [14,15]. Attempts have also been made to expand the knowledge of the genotypic and phenotypic diversity of *G. vaginalis* strains in terms of virulence factors: particularly vaginolysin, sialidase, and biofilm-forming proteins [16-18]. The development of methods for the genotypic differentiation of *G. vaginalis* revealed that the genomes exhibit great variability. Therefore, some conventional methods, including pulse field gel electrophoresis, restriction fragment length polymorphism, classical ribotyping with Southern blot, and restriction enzyme analysis, are not applicable for typing this species [19-21]. The amplified ribosomal DNA restriction analysis method, while applicable to the genotypic differentiation of *G. vaginalis*, has been found to not be discriminatory enough for pathogenetic and epidemiological studies of *G. vaginalis*[17,18].

Recent data from *G. vaginalis* comparative genomic analyses have indicated that the bacterium possesses a small core genome, consisting of 746 genes, that accounts for only 27% of the pan-genome of the species [22]. The small number of unique genes (21) in the individual strains of *G. vaginalis* and the genomic plasticity among the strains suggest that horizontal gene transfer (HGT) is active; but there is frequent homologous recombination among *G. vaginalis* strains, as well as the intake of foreign DNA from other species [15,22].

Clustered Regularly Interspaced Short Palindromic Repeats (CRISPRs) and their associated *cas* genes constitute a bacterial and archaeal defence mechanism against exogenous nucleic acids [23]. The majority of archaea and approximately half of bacterial genomes contain CRISPR loci [24]. CRISPR loci consist of unique sequences (spacers) that intercalate between short conserved repeat sequences. The spacer sequences often originate from invading viruses and plasmids [25,26]. The CRISPR/Cas defence mechanism relies on RNA interference that prevents bacteriophage infection and plasmid conjugation, thus restricting two routes of HGT [27]. Analyses of CRISPR sequences have been used in a variety of applications including strain genotyping and epidemiological study, detection of evolutionary events and bottlenecks, investigation of the history of virus exposure, and host population dynamics, providing insights into the dominant routes of HGT [28-32]. The current study targeted the detection and analysis of CRISPR loci in the genomes of 17 *G. vaginalis* strains isolated from the vaginal tracts of women diagnosed with BV [18], and also in the genomes of 21 *G. vaginalis* strains deposited in the NCBI genome database.

In the current study, we examined the origins of CRISPR spacers representing the immunological memory of *G. vaginalis* strains, and we hypothesised about the impact of CRISPR/Cas on the emergence of genetic variability of *G. vaginalis* strains. Also, we demonstrated the restricted distribution of the CRISPR loci among the *G. vaginalis* strains.

## Methods

### *G. vaginalis* strains

Seventeen *G. vaginalis* strains isolated from clinical specimens obtained from the vaginal tracts of women diagnosed with BV were used in this study [18]. The isolates had been previously genotyped/biotyped and characterised with respect to the main known virulence factors, namely vaginolysin and sialidase [18].

Three completely sequenced *G. vaginalis* genomes (ATCC14019, CP002104.1; 409–05, CP001849.1; and HMP9231, CP002725.1) and 18 *G. vaginalis* draft genomes were retrieved from the NCBI genome database (http://www.ncbi.nlm.nih.gov/genome/genomes/1967). The accession numbers of the draft genomes are listed in Additional file 1.

### CRISPR amplification and sequencing

Primers for CRISPR amplification were designed by genomic comparison of the CRISPR flanking regions of *G. vaginalis* strains ATCC 14019, 5–1, AMD, 409–05, 41V, 101, and 315A. Three different sets of primers; Cas-1-1fw, Cas-3-1fw, CR-1rev, CR-2rev and CR-3rev; were used for the amplification of the CRISPR regions (Additional file 2). PCR was performed in a 50-μl reaction mixture containing 0.2 μM each primer, 20 ng genomic DNA and 1.5 U Long PCR Enzyme Mix (Thermo Scientific Fermentas, Vilnius, Lithuania). The reaction mixture was subjected to 28 cycles of denaturation at 94°C for 30 s, primer annealing at 50°C for 40 s, and extension at 72°C for 3 min. The final extension step was prolonged to 10 min. PCR products were purified using GeneJET Gel Extraction Kit (Thermo Scientific Fermentas) according to the manufacturer‘s instructions. The cloned DNA fragments were subjected to sequencing using the ABI 3130XL genetic analyser. Sequence walking was explored using internal primers constructed within the spacer sequences to complete the sequencing of the PCR fragments.

A slightly modified spacer-crawling approach [29] was applied to amplify the CRISPR arrays of strains GV28 and GV33. The primers targeted *cas2* and the repeat sequence within the CRISPR locus. The resulting PCR product represented a ladder consisting of a number of fragments with increasing lengths: each fragment differed by the length of one spacer and one repeat. The mixture of fragments was cloned into the pJET1.2 vector (Thermo Scientific Fermentas); the recombinant plasmids containing the longest DNA inserts were selected and then subjected to sequencing. The next round of amplification used the primer generated from the further spacer sequence and the primers located on the flanking regions downstream of the CRISPR sequence (Additional file 2). The resulting contigs were assembled with a minimum overlapping region of three spacers.

### Amplification and sequencing of the *cas* genes

The presence of the *cas* genes was verified by amplification of the regions containing *cas5*-*cas6e*-*cas1*-*cas2* (~3.6 kbp), *cas3*-*cse1* (~3 kbp), *cse2*-*cas5* (~2.7 kbp), *cas5* (~0.88 kbp) and *cse2* (~0.6 kbp). The primers used in the PCR are provided in Additional file 2. The PCR regimen included 28 cycles of denaturation at 94°C for 30 s, primer annealing at 58°C for 30 s, and extension at 72°C for 1 min/kb PCR target. The final extension step was prolonged to 10 min. The cloned DNA fragments containing *cas5* and *cas2* were subjected to sequencing.

### CRISPR sequence analysis

CRISPR information for the three *G. vaginalis* genomes (ATCC14019, 409–05, and HMP9231) was retrieved from the CRISPR database [24]. CRISPRs Finder [24] was used to detect CRISPR repeat and spacer sequences. The identification of *cas* genes was also performed using NCBI BLAST (http://blast.ncbi.nlm.nih.gov/Blast.cgi). Each piece of CRISPR and *cas* information retrieved from the databases was manually proofread. The search for similarities between each spacer and the sequences deposited in GenBank was performed using BLASTn at NCBI, with the search set limited to Bacteria (taxid:2) or Viruses (taxid:10239). All matches with a bit score above 40.0, corresponding to 100% identity over at least 20 bp, were considered legitimate hits. Only the top hit was taken into consideration. Matches to sequences found within *G. vaginalis* CRISPR loci were discarded. Spacers were compared to one another using the MAFFT program [33]. CRISPR spacers with up to three mismatches that had 100% overlap between sequences were considered identical. The consensus sequences of the CRISPR repeat and protospacer region alignments were generated by WebLogo [34].

## Results

### Architecture of CRISPR/Cas loci in *G. vaginalis* strains

**Figure 1:**
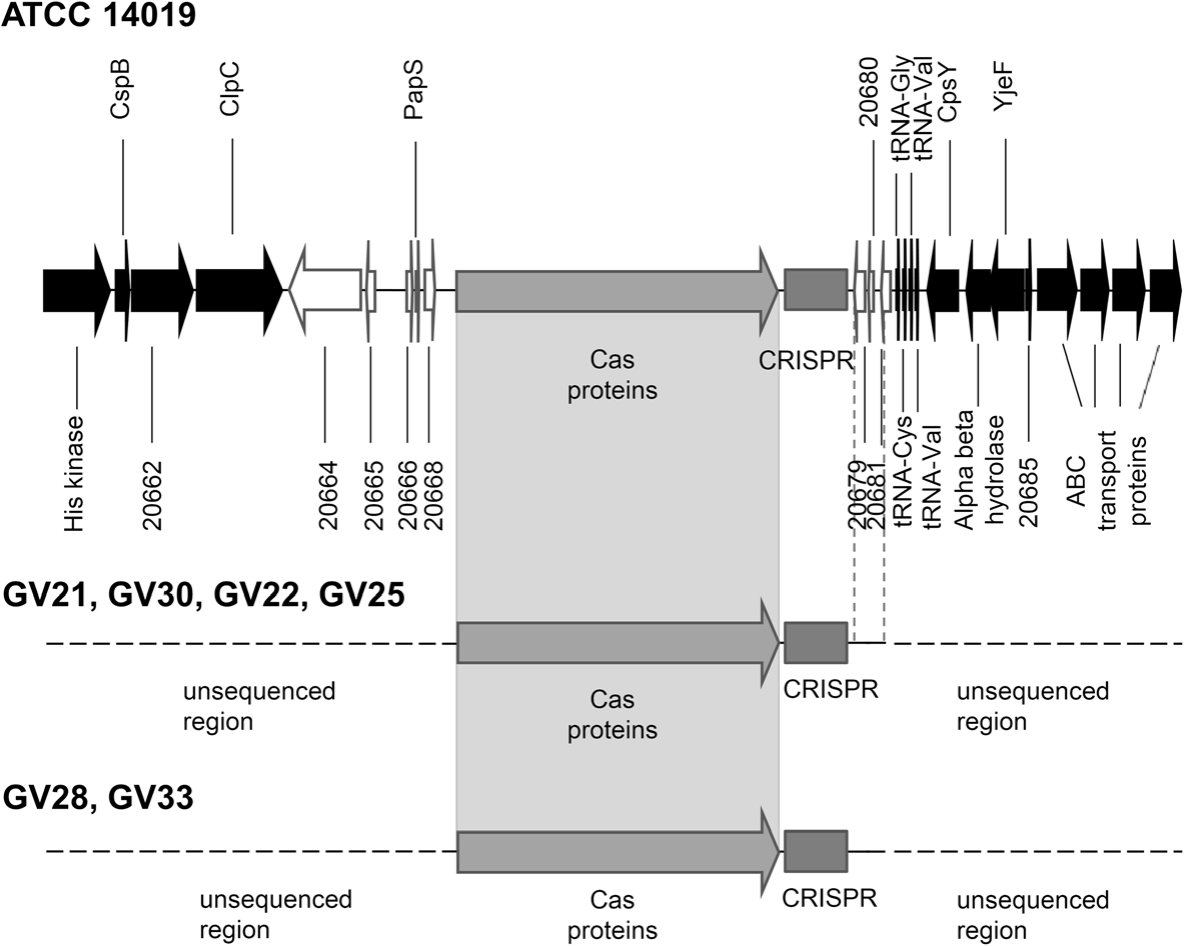
**Position of CRISPR/Cas locus on the chromosome of *G. vaginalis*.** The flanking sequence region shared by several strains downstream of the CRISPR array is marked by vertical dashed lines.

Two of the three completely sequenced *G. vaginalis* genomes, 12 of the 18 draft genomes in GenBank, and 6 of the 17 *G. vaginalis* clinical isolates contained a *cas* gene cluster and a CRISPR locus. Sequences consisting of repeats/spacers adjacent to the *cas* genes were considered CRISPR sequences. The CRISPR/Cas loci in the majority of strains were located between the core gene *clpC* and the gene encoding tRNA_Gly_ (Figure 1).

The region between the 3^′^-end of *clpC* and the *cas* genes had ORFs encoding hypothetical proteins and was variable in length (~5-19 kbp), depending on the strain. The region between the 3^′^-end of the CRISPR array and the gene encoding tRNA_Cys_ was not conserved among *G. vaginalis* strains and varied in length (0.4-1.8 kbp) from strain to strain. The CRISPR/Cas loci of strains 409–05, 00703B, and 00703C2 had different flanking sequences surrounding them. Notably, the region downstream of the CRISPR arrays found in clinical isolates GV21, GV30, GV22, and GV25 corresponded to that found in the genome of the ATCC14019 strain; while the CRISPR flanking sequences on the right, determined in the GV28 and GV33 strains, did not show any similarity to the sequences detected downstream of the *G. vaginalis* CRISPRs. Due to the variability of the flanking sequences downstream of the CRISPR locus and long CRISPR amplicon, strains GV28 and GV30 contained *cas* genes but did not produce PCR products. The CRISPR sequences in those two strains were identified using the spacer-crawling approach described in the Methods section. The sequences of the amplified CRISPR regions of six *G. vaginalis* strains analysed in this study were deposited to GenBank database under the Accession numbers JX215337-JX215342.

The *cas* loci of *G. vaginalis* consisted of the *cas* genes *cas3cse1cse2cse4cas5cas6ecas1cas2*. The detected gene cluster belongs to type I, subtype I-E, known as Ecoli [35]. CRISPR loci were located downstream of *cas2* and contained from 1 to 50 spacer sequences. Amplification of the regions containing different *cas* genes was performed to eliminate false-negative PCRs for CRISPR sequences. PCR products consisting of different sets of *cas* genes (*cas5cas6ecas1cas2*, *cas3cse1, cse2cas5, cas5,* and *cas2*) were obtained from clinical isolates identified as being PCR-positive for CRISPR sequences. The sequences of *cas2* and *cas5* were subjected to sequencing, and their sequences were deposited in GenBank under the Accession numbers JX215343-JX215345.

### Characterisation of CRISPR repeat and spacer sequences

**Figure 2:**
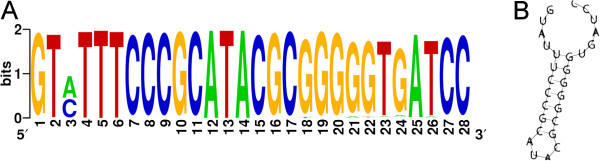
**Features of the repeat in the *G. vaginalis *CRISPR arrays.** (**A**) Sequence logo for all repeats in the CRISPR loci of *G. vaginalis.* The height of the letters shows the relative frequency of the corresponding nucleotide at that position. (**B**) Secondary structure of the *G. vaginalis* repeat region predicted using RNAfold [36] .

The repeat sequence found in the CRISPR loci of the 20 *G. vaginalis* strains consisted of 28 bp (Figure 2A), while the spacers in the loci varied in size from 33 to 34 bp. The most variable nucleotide positions were found at the proximal ends of the CRISPR repeat (Figure 2A). The repeat sequence of CRISPR was partially palindromic and forms a putative RNA secondary structure with ΔG < − 10 kcal/mol (Figure 2B).

**Figure 3:**
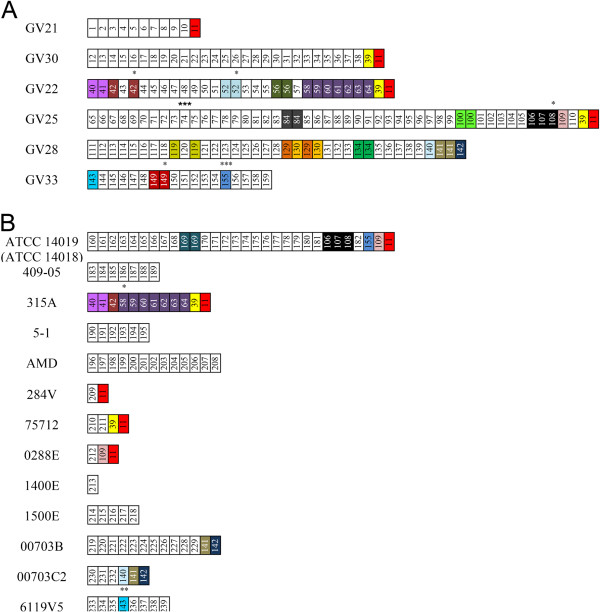
**Graphic representation of CRISPR spacers in *G. vaginalis *clinical isolates (A) and *G. vaginalis *genomes deposited in GenBank (B).** Spacers are represented by boxes; repeats are not included. The leader-end spacers are oriented on the left of each array; the trailer-end spacers are oriented on the right side of each array. Identical spacers are represented by the same number and colour. Unique spacers are white-coloured. Spacers with mismatches of up to three nucleotides (see Methods) are indicated by dots on the top of the spacer. The number of dots shows the number of mismatched nucleotides.

The CRISPR arrays found in the *G. vaginalis* strains varied in length and spacer content: the longest CRISPR locus contained 40 unique spacers (40/50) and was detected in clinical isolate GV25, while only one spacer adjacent to the *cas* genes was found in strain 1400E. Across six clinical isolates of *G. vaginalis*, 175 spacers were identified; among them, 129 unique spacers were detected (Figure 3). The fourteen *G. vaginalis* genomes deposited in GenBank carried 81 unique spacers out of the 110 spacer sequences that were analysed (Figure 3). A total of 285 spacers adjacent to the *cas* genes were identified among the 20 *G. vaginalis* strains containing CRISPR/Cas loci (Figure 3).

The trailer-end spacers of the CRISPR loci, i.e. the oldest spacers found farthest from the leader sequences [37], exhibited several types of conservation: nine strains of *G. vaginalis* shared one spacer, five strains (among them, the three clinical isolates GV22, GV25, and GV30) shared two spacers, whereas three strains (GV28, 00703B and 00703C2) contained distinct spacer sequence conservation at the trailer -end (Figure 3). All spacer sequences detected within the CRISPR locus of *G. vaginalis* strain 315A had a copy at the trailer-end of clinical isolate GV22 (Figure 3).

### Analysis of CRISPR spacer sequences

**Figure 4:**
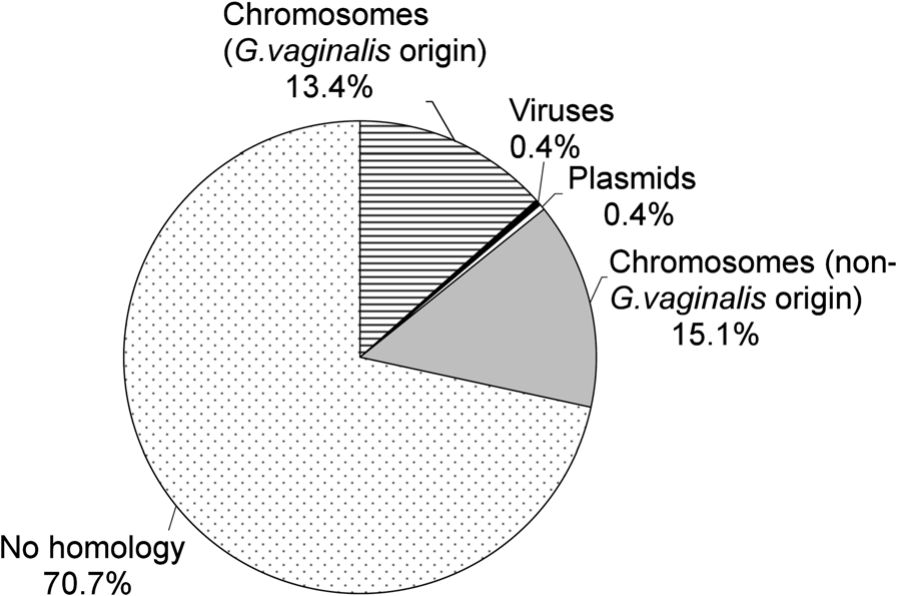
**Matches of CRISPR spacers identified in *G. vaginalis *strains to plasmid, bacteriophage, and chromosomal sequences, expressed in percentages.**

All 210 unique spacer sequences were blasted against phage, plasmid, and bacterial sequences. It has been suggested that 100% identity between spacer and protospacer sequences is required to provide CRISPR-mediated immunity [38]; while the tolerance for mismatches is not yet completely elucidated [39,40]. Therefore, a search for protospacers was performed, exploring a less stringent identity criterion by setting a cut-off described in the Methods section. A total of 70.7% of the spacers had no match to the GenBank database (Figure 4). Overall, among the 70 spacers with matches to the selected cut-off, one sequence showed similarity to a viral sequence, one sequence matched a plasmid sequence, and 68 sequences (97%) showed similarity to chromosomal sequences (Figure 4; Additional file 3). Among the CRISPR spacers matched to chromosomal sequences of non-*G.vaginalis* origin, five of 77 spacers were similar to sequences originating from human-associated bacteria including *Haemophilus influenza*, *Weeksella virosa*, *Campylobacter jejuni*, and *Bacillus cereus* (Additional file 3B). Nearly half of the spacers (32 of 77) were similar to *G. vaginalis* chromosomal sequences, including 10 spacers that shared 100% identity (33 of 33 nucleotides; Additional file 3A). All of these spacers, almost uniformly distributed throughout the CRISPR arrays, were unique sequences except for spacer #106 located at the CRISPR trailer-end of strains ATCC14019, ATCC 14018, and GV25.

### Spacers matching *G. vaginalis* chromosomal sequences

The 28 spacers had significant nucleotide matches to *G. vaginalis* chromosomal regions (85 to 100% identity), except for three spacers in the CRISPR array of strain 00703B and one spacer found in strain GV22 displaying up to 77% identity (Additional file 3A). Few spacers shared identity with the sequences annotated as having phage origin. Analysis of the *G. vaginalis* genomes revealed the existence of four to seven phage-associated genes, though most of those were present in one strain and absent in the other strains [15]. We were not able to determine whether the clinical isolates contained the sequences of phage origin targeted by the spacers, because the complete genome sequences are not available yet.

A majority of the spacer hits that mapped to the sequences did not associate with mobile elements (Additional file 3A). The protospacers are localised on both strands of the *G. vaginalis* chromosome, covering coding and non-coding regions. A substantial number of spacers targeting the same region were distributed consecutively in the CRISPR arrays. Nearly 60% of the CRISPR spacers targeted protospacers located on the chromosome of *G. vaginalis* strain 409–05 (Additional file 3A). Moreover, different spacers from the CRISPR arrays of different strains targeted the same region of the chromosome. Namely, the spacers in the CRISPR arrays of strains GV22 (one spacer), GV25 (one spacer), GV28 (one spacer), and GV30 (five spacers) clustered in a small 1.1 kbp area and matched the same non-coding region on the chromosome of strain 409–05 (Additional file 3). We did not identify spacers in the CRISPR array of strain 409–05 that shared homology with regions of *G. vaginalis* chromosomal DNA. Several spacers (#100 and #163) originating from different strains targeted the gene encoding N-acetylmuramoyl-L-alanine amidase. None of the CRISPR spacers were found to be self-targeting. We examined the five genomes of *G. vaginalis* available in the NCBI genome database that had spacers targeting coding and non-coding regions on the chromosomes of strains 409–05, 6420B, 315A, 41 V, ATCC14019, and AMD. We did not find a match between the spacers and the endogenous genomic sequences, except for the sequences located in the CRISPR arrays.

**Figure 5:**
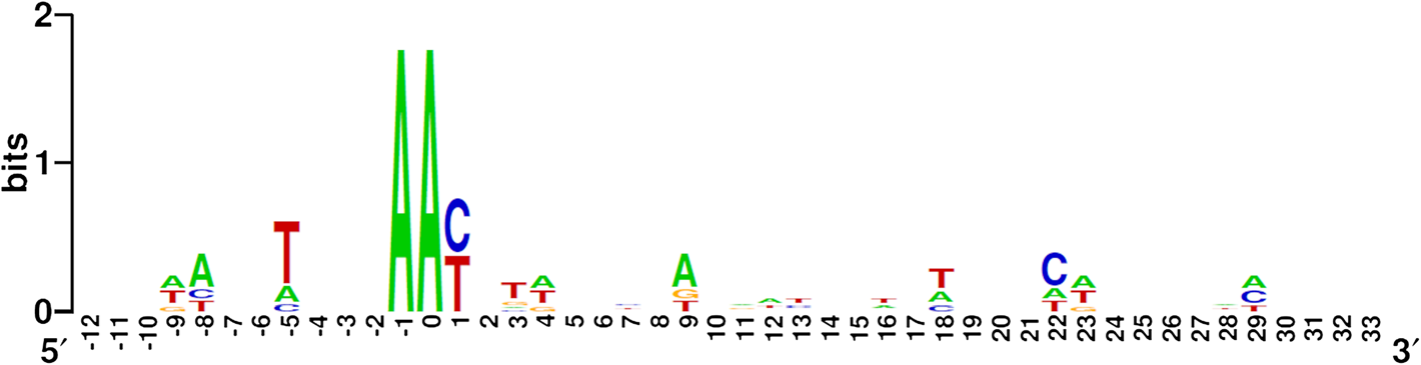
**WebLogo for the PAM consensus sequence determination.** Ten protospacers identical to spacers were aligned relative to the 5^′^-end of the protospacer (base 1). Sequences include the protospacer (positive numbers) and 13 nucleotides (negative numbers) upstream of the first base of the protospacer (containing the PAM).

We also analysed whether the protospacers located on the *G. vaginalis* chromosome displayed conserved protospacer adjacent motif (PAM) sequences [41,42]. We aligned the protospacers with the flanking regions comprising 20 bp on both sides. Alignments were performed for ten protospacers sharing 100% identity with the spacers. The conserved motif of two nucleotides (AA) situated immediately upstream of the target region was detected (Figure 5). The PAM signature AA was confirmed for nine protospacers with up to 10% mismatches located distant from the 5^′^- and 3^′^-ends of the spacers.

Thus, the motifs adjacent to the protospacers located in the *G. vaginalis* genomic DNA bear the signatures of PAMs. The orientation of the *G. vaginalis* PAM is 5^′^-AA-protospacer-3^′^, which coincides with the orientation of the PAM identified in *E. coli* as CRISPR/Cas; both bacteria belong to the same type [41,42]. Among all of the *G. vaginalis* CRISPR arrays, the first nucleotide of 97.5% of the spacers was either C or T. Only six spacers started with A or G (2.5%). All of the spacers targeting the protospacers on the *G. vaginalis* chromosome started with C or T (18:13).

## Discussion

The CRISPR locus of the recently discovered CRISPR/Cas defence system in prokaryotes protects against invading viruses and plasmids and is a map of the “immunological memory” of the microorganism [25,26]. The spacer sequences that are incorporated into the CRISPR loci provide a historical view on the exposure of the bacteria to a variety of foreign genetic elements [23]. A recent report on the ability of CRISPR/Cas to prevent natural transformation in *Streptococcus pneumoniae* enlarged the role of CRISPR in bacterial nucleic acid-based immunity and the impact that CRISPR has on the emergence of bacterial pathogens [43].

In the current study, we analysed the CRISPR arrays in 17 recently characterised *G. vaginalis* clinical isolates [18] and the genomes of 21 of *G. vaginalis* strains deposited in the NCBI genome database. We examined the spacer repertoire and evaluated the potential impact of CRISPR/Cas on gene uptake in *G. vaginalis*.

We found that six clinical isolates (35%) and 14 *G. vaginalis* genomes deposited in the NCBI database (67%) contained CRISPR/Cas loci. The loci included complete *cas* genes and repeat sequences interspaced by a variable number of spacers. The repeat sequence in the CRISPR array of *G. vaginalis* was not identical to that found in the *E. coli* CAS-E subtype [44]. *In silico* analysis of the Cas proteins revealed highly conserved (>97% identity) sequences among the *G. vaginalis* strains. The Cas proteins showed the highest similarity (46 to 63% identity) to the proteins from *A. vaginae* DSM15829 (Ecoli Cas subtype); meanwhile, 9 to 35% identity was scored to the Cas proteins from *E. coli* K12 strain MG1655, which are attributable to the same subtype [35]. The AT-rich leader sequence immediately upstream of the first CRISPR repeat was detected in the genomes of all of the analysed *G. vaginalis* strains.

Analysis of the spacer repertoire revealed different activities of the CRISPR/Cas loci across different *G. vaginalis* strains. The CRISPR locus identified in the genome of strain GV25 is considered to be the most active, in terms of the degree of spacer polymorphism exhibited by both the total number of unique spacers and the total number of unique spacer arrangements [38,45]. In contrast, the spacer content in the CRISPR array of strain 315A could indicate that newly gained CRISPR spacers were deleted and the most ancient spacers were preserved (Figure 3B). We may assume that *cas* activity in the genome of *G. vaginalis* strain 315A was depleted [37,45].

In the present work, the analysis of CRISPR loci revealed that the majority of CRISPR spacers were similar to chromosomal sequences of both *G. vaginalis* and non-*G.vaginalis* origins. Spacer matches to viral and plasmid sequences suggest their putative origin, because there is no evidence of plasmids in the *G. vaginalis* genomic architecture, and viruses that infect *G. vaginalis* are not yet known [15,22]. A substantial portion of the spacers matched *G. vaginalis* chromosomal sequences. The spacers shared identity with coding and non-coding sequences in the chromosome of *G. vaginalis*. The spacers were not self-targeting [46], and the protospacers located on the chromosome displayed PAMs. The question of whether C or T is the first base of the spacer or the 29th base of the repeat in *G. vaginalis* CRISPR arrays is still open [46,47]. In our study, all spacers targeting protospacers on the *G. vaginalis* chromosome started with either C or T. Thus, the spacers correspond to the AAT-PAM or AAC-PAM, assuming that the C/T originates from the repeat. Hypotheses about the borders of the CRISPR repeats/spacers need experimental testing; however, the idea of a “duplicon” seems attractive [47].

The analysis of the genomes of *G. vaginalis* presumed that the chromosomal sequences targeted by spacers did not derive from plasmids or viruses and that the genes in the vicinity of the protospacers (approx. 5 kbp upstream and 5 kbp downstream) do not have viral origin. The gene-coding sequences targeted by the *G. vaginalis* CRISPR/Cas system were found to not be constituents of mobile-element-associated genes such as restriction-modification and toxin-antitoxin systems or transposases [45,48]. Two spacers from different strains targeted the gene encoding N-acetylmuramoyl-L-alanine amidase: a CHAP-family domain protein found to have lytic ability [49]. Several strains possess spacers matching the gene encoding the glycoside hydrolase (GH) family 25 protein and the non-coding regions in its close vicinity. The GH 25 family comprises lysozyme able to hydrolyse peptidoglycan and two Abi proteins conferring resistance to a broad range of related bacteriocins [15,50]. It has been suggested that these findings are in agreement with the data showing that *G. vaginalis* strains produce substances antagonistic to bacterial isolates common to the vaginal microbiome [15,51]. A substantial part of the spacers targeted non-coding regions or ORF’s encoding hypothetical proteins with undefined functions.

Our data suggest that the CRISPR/Cas system was in touch with *G. vaginalis* DNA that was most probably of chromosomal origin and accessed by the transformation, transduction, or conjugation routes. DNA acquisition and exchange by natural transformation among *G. vaginalis* strains was detected as a favourable route [22]. Moreover, *G. vaginalis* strains were found to encode the competence promoting proteins ComEA, ComEC, and CinA [15]; http://blast.ncbi.nlm.nih.gov/Blast.cgi. Our data on the origin of the spacers detected in the *G. vaginalis* CRISPR arrays propose the hypothesis that the transfer of genetic material among *G. vaginalis* strains could be regulated by the CRISPR/Cas mechanism. Circumstances favourable for DNA transfer and CRISPR activity would mean the simultaneous presence of more than one *G. vaginalis* strain during infection, which is consistent with previous reports [21,22,52]. The impact of CRISPR/Cas on the virulence of *G. vaginalis* could involve the spacer targeting the GH family 25 gene that encodes a product promoting competitive exclusion by the 409–05 strain http://blast.ncbi.nlm.nih.gov/Blast.cgi.

The distribution of CRISPR/Cas loci among pathogenic bacteria that incorporate new genetic material, along with virulence genes, through natural transformation is variable [27,43]. The incidence of the CRISPR/Cas system among *G. vaginalis* strains may be determined by the habitat of the bacteria. The low prevalence of viruses in the human endometrium [53] does not promote the acquisition of CRISPR/Cas by *G. vaginalis* as an adaptive immunity system against foreign DNA. However, the human vagina is a more favourable environment for virus progression, and extravaginal reservoirs have an impact on the distribution of viruses in the vaginal tract [54]. Recent papers have demonstrated that pathogenic bacteria may lose CRISPR/Cas under certain selective pressure [55,56]. The presence of multiple antibiotic resistances is correlated with the loss of CRISPR loci in enterococci [55]. However, we did not find a correlation between the presence of CRISPR/Cas loci and genes responsible for antibiotic/antimicrobial resistance in *G. vaginalis* strains. *In silico* analysis of *G. vaginalis* genomes revealed that strains 14018, 14019, 284 V, 315A, 1400E, 0288E, and 00703B, all of which possessed CRISPR/Cas, contained genes conferring resistance to bleomycin and methicillin [15]; http://blast.ncbi.nlm.nih.gov/Blast.cgi. In addition, *G. vaginalis* strains 14018 and 14019 contained a gene coding for an aminoglycoside phosphotransferase that increased resistance to aminoglycosides [15]. Selective pressure for virulence other than antibiotic resistance might also have an impact on the presence of CRISPR/Cas loci. In our study, however, the distribution of CRISPR/Cas systems was variable among the *G. vaginalis* strains with elevated virulence potential that were isolated from BV patients (Table 1). Thus, our results did not reveal a potential link between the presence of CRISPR loci and the known virulence features of the strains (Table 1). Overall, our data suggest that CRISPR-based typing does not provide a promising tool for epidemiological discrimination of *G. vaginalis* strains. Moreover, *G. vaginalis* genomic DNA has exhibited such a great variability [19-22] that the possibility of developing a routine PCR using a set of specific primers for CRISPR loci amplification is doubtful.

**Table 1:** ***G. vaginalis *CRISPR spacers and known virulence features**

**Strain**	**Reference**	**Clinical status**	**Biotype**	**Sialidase A**		**Vaginolysin**		**CRISPR**	
				**Coding gene**	**Activity**	**Coding gene**	**Production level**	**Number of spacers**	**Number of unique spacers**
ATCC 14019	[15]	BV	ND	+	ND	+	ND	30	24
ATCC 14018	[15]	BV	1	-	-	+	ND	30	24
409-05	[15]	Asymptomatic BV	ND	-	-	+	ND	7	7
HMP9231	CP0027525.1	Not known	ND	+	ND	+	ND	-	-
101	[14]	BV	ND	+	ND	+	ND	-	-
41V	AEJE01000000.1	Healthy woman	ND	+	ND	+	ND	-	-
315A	AFDI01000000.1	Not known	ND	+	ND	+	ND	11	0
5-1	[14]	Healthy woman	ND	-	-	+	ND	6	6
AMD	[14]	BV	ND	-	-	+	ND	13	13
284V	[22]	Abnormal discharge & odor	1	+	ND	+	ND	2	1
75712	[22]	BV	1	+	ND	+	ND	3	2
0288E	[22]	Abnormal discharge & odor	1	+	ND	+	ND	3	1
6420LIT	[22]	Healthy woman	2	-	-	+	ND	-	-
6420B	[22]	Healthy woman	2	-	-	+	ND	-	-
55152	[22]	Asymptomatic BV	3	+	ND	+	ND	-	-
1400E	[22]	Nugent score 9	4	+	ND	+	ND	1	1
1500E	[22]	Nugent score 7	5	+	ND	+	ND	5	5
00703Bmash	[22]	BV	2 or 5	+	ND	+	ND	13	11
00703C2mash	[22]	BV	2 or 5	+	ND	+	ND	6	3
00703Dmash	[22]	BV	3 or 7	+	ND	+	ND	-	-
6119V5	[22]	Nugent score 5	7	+	ND	+	ND	8	7
GV15	[18]	BV	5	+	S	+	Low	-	-
GV17	[18]	BV	5	+	S	+	Low	-	-
GV21	[18]	BV	1	+	W	+	Medium	11	10
GV22	[18]	BV	2	+	-	+	Low	30	13
GV23	[18]	BV	1	+	W	+	High	-	-
GV24	[18]	BV	1	+	-	+	Low	-	-
GV25	[18]	BV	1	+	W	+	Low	50	40
GV26	[18]	BV	1	+	-	+	Low	-	-
GV28	[18]	BV	5	+	S	+	High	37	25
GV29	[18]	BV	1	+	-	+	Low	-	-
GV30	[18]	BV	1	+	-	+	Low	29	27
GV31	[18]	BV	1	+	W	+	Medium	-	-
GV32	[18]	BV	1	+	-	+	Medium	-	-
GV33	[18]	BV	5	+	S	+	Low	18	14
GV34	[18]	BV	4	+	-	+	Low	-	-
GV35	[18]	BV	5	+	S	+	Low	-	-
GV36	[18]	BV	2	+	S	+	Medium	-	-

ND – not done.

The fact that the majority of *G. vaginalis* strains analysed so far were isolated from symptomatic and asymptomatic BV patients, while only few strains were obtained from the vaginas of healthy women, could be an impetus moving forward to elucidate a link between commensal *G. vaginalis* strains and CRISPR/Cas loci (Table 1). Recent findings on the role of Cas proteins in providing adaptive immunity to bacteria [39,43,57] may motivate experimental testing of hypotheses on how CRISPR/Cas impacts the regulation of the transfer of genetic material among *G. vaginalis* strains.

The present study is the first attempt to determine and analyse CRISPR loci in bacteria isolated from the human vaginal tract. The relationship between prokaryotes and their environment that is recorded in the spacer sequences of CRISPR loci sheds light into the genomic evolution of *G. vaginalis*.

## Conclusions

The CRISPR/Cas system was detected in the genomes of about one- half of the analysed *G. vaginalis* strains. The *cas* genes in the CRISPR/Cas loci of *G. vaginalis* belong to the Ecoli subtype. A total of 285 spacers adjacent to the *cas* genes were identified among the 20 *G. vaginalis* strains containing CRISPR/Cas loci. Approximately 20% of all of the spacers in the CRISPR arrays had matches in the GenBank database. Sequence analysis of the CRISPR arrays revealed that nearly half of the spacers matched *G. vaginalis* chromosomal sequences. The spacers sharing identity with these chromosomal sequences were determined to not be self-targeting, and presumably were neither a constituent of mobile-element-associated genes nor originated from plasmids/viruses. The spacer hits were mapped to *G. vaginalis* chromosomal genes, non-coding regions, or ORF’s encoding hypothetical proteins with undefined functions. The protospacers located on the *G. vaginalis* chromosome display conserved PAMs. We did not find a link between the presence of CRISPR loci and the known virulence features of *G. vaginalis*. Based on the origin of the spacers found in the *G. vaginalis* CRISPR arrays, we hypothesise that the transfer of genetic material among *G. vaginalis* strains could be regulated by the CRISPR/Cas mechanism. Our findings may provide deeper insights into the genetics of *G.vaginalis* and promote further studies on the role of *G. vaginalis* in the microbiome of its host.

## Competing interests

The authors declare no competing interests.

## Authors’ contributions

MP carried out primer design and all DNA manipulation procedures, analyzed all results and drafted the manuscript. MZ performed all bioinformatic analysis of CRISPR/Cas system in *G. vaginalis* genomes. AZ participated in the design of the study and revised the manuscript. All authors read and approved the final manuscript.

## Supplementary Material

Additional file 1 **Accession numbers of the draft genomes of *G. vaginalis* strains used in the study.** Click here for file

Additional file 2 **Primers used for CRISPR loci and*cas*genes amplification.** Click here for file

Additional file 3 **A.Analysis of CRISPR spacers matched to chromosomal sequences of*G. vaginalis*origin.B.** Analysis of CRISPR spacers matched to chromosomal sequences of non-*G.vaginalis* origin. The table contains information on the spacer sequences retrieved from the amplified CRISPR loci of *G. vaginalis* clinical isolates and from *G. vaginalis* genomes deposited in GenBank. The analysis of spacer hits mapped to chromosomal sequences of *G. vaginalis* and non-*G. vaginalis* origin are provided. Click here for file
