# Supplementary material for: Insights into the CRISPR/Cas system of Gardnerella vaginalis
Source: BMC Microbiol. 2012 Dec 21;12:301. doi: 10.1186/1471-2180-12-301 (PMC3559282; doi:10.1186/1471-2180-12-301)
Supplement: Additional file 1 — Accession numbers of the draft genomes of G. vaginalis strains used in the study. [file 1471-2180-12-301-S1.docx]

**Additional file 1. Accession numbers of the draft genomes of *G. vaginalis* strains used in the study**

| *G. vaginalis* strain | Accession no. |
| --- | --- |
| 101 | AEJD00000000.1 |
| 41V | AEJE00000000.1 |
| 315A | AFDI00000000.1 |
| 5-1 | ADAN00000000.1 |
| AMD | ADAM00000000.1 |
| ATCC 14018 | ADNB00000000.1 |
| 284V | ADEM00000000.1 |
| 75712 | ADEM00000000.1 |
| 0288E | ADEN00000000.1 |
| 6420LIT | ADEO00000000.1 |
| 6420B | ADEP00000000.1 |
| 55152 | ADEQ00000000.1 |
| 1400E | ADER00000000.1 |
| 1500E | ADES00000000.1 |
| 00703Bmash | ADET00000000.1 |
| 00703C2mash | ADEU00000000.1 |
| 00703Dmash | ADEV00000000.1 |
| 6119V5 | ADEW00000000.1 |
