# Supplementary material for: Insights into the CRISPR/Cas system of Gardnerella vaginalis
Source: BMC Microbiol. 2012 Dec 21;12:301. doi: 10.1186/1471-2180-12-301 (PMC3559282; doi:10.1186/1471-2180-12-301)
Supplement: Additional file 2 — Primers used for CRISPR loci andcasgenes amplification. [file 1471-2180-12-301-S2.docx]

**Additional file 2. Primers used for CRISPR loci and *cas* genes amplification**

| Amplified DNA fragment | Primer name | Primer sequence (5’-3’) |
| --- | --- | --- |
| CRISPR | Cas-1-1fw† | TAATAGATTGCGAAACTACAGG |
| CRISPR | Cas-1-2fw | GCGAAACTACAGGTTTAAATAGCA |
| CRISPR | Cas-3-1fw | AATTGCGATAGCGTTAGTAATTTT |
| CRISPR | CR-1rev‡ | CCCACCGTAAGGTCGAATAA |
| CRISPR | CR-2rev | CCCGAAATATTCTCAAATGC |
| CRISPR | CR-3rev | GAGTGGGCGATACAAGGTTC |
| CRISPR, strains GV28 and GV33 | CR-4rev | CCACCACTAGACTACTACAACC |
| CRISPR, strains GV28 and GV33 | CR-5rev | CGCTGGCATTGGATTCACTC |
| CRISPR, strains GV28 and GV33 | CR-6rev | CCGCGTTTTTAGGGTGCTT |
| CRISPR, strains GV28 and GV33 | CR-7rev | CCACACTTGCCGCACATG |
| *cas5-cas6e-cas1-cas2* genes | Cas5fw | CATATGAAAAGTTTATTACTGAAGTTTTCTGG |
|  | Cas2rev | ACCCATTGTTGCACACAATCC |
| *cas5*gene | Cas5fw | CATATGAAAAGTTTATTACTGAAGTTTTC |
|  | Cas5rev | CTCGAGTTAAGACTCCAATCCAGAAAAAAGC |
| *cse2* gene | Cse2fw | ATGCTTGCTCAACTACGTCACAC |
|  | Cse2rev | GCGATAATATTCTTGCGCCCAG |
| *cas3-cse1*genes | Cas3fw | ATGAGTTGTAATCACGTTGTTAAT |
|  | Cse1rev | TGAGTTTTCATATCGCCAGC |

^†^fw – forward primer

^‡^rev – reverse primer
